# Is COPD associated with increased risk for microaspiration in intubated critically ill patients?

**DOI:** 10.1186/s13613-020-00794-1

**Published:** 2021-01-11

**Authors:** Thècle Degroote, Emmanuelle Jaillette, Jean Reignier, Farid Zerimech, Christophe Girault, Guillaume Brunin, Arnaud Chiche, Jean-Claude Lacherade, Jean-Paul MIRA, Patrice Maboudou, Malika Balduyck, Saad Nseir, for the MicroCOPD study group, Emmanuelle Mercier, Pierre-Louis Declercq, Michel Sirodot, Gaël Piton, François Tinturier, Elisabeth Coupez, Stéphane Gaudry, Michel Djibré, Didier Thévenin, Céline Broucqsault-Dedrie, Stéphanie Barrailler, Cyril Fayolle, Franck Minacori, Isabelle Alves

**Affiliations:** 1 Service de Médecine Intensive et Réanimation, Groupe Hospitalier Paris Saint-Joseph, Paris, France; 2 Critical Care Center, CHU Lille, 59000 Lille, France; 3 Medecine Intensive Réanimation, Centre Hospitalier Universitaire de Nantes, Nantes, France; 4 Université de Nantes, Nantes, France; 5 Centre de Biologie Et de Pathologie, CHU Lille, 59000 Lille, France; 6 Normandie Univ, UNIROUEN, EA 3830, Rouen University Hospital, Medical Intensive Care Unit, 76000 Rouen, France; 7 Intensive Care Unit, Boulogne Sur Mer Hospital, Boulogne-sur-Mer, France; 8 Intensive Care Unit, Tourcoing Hospital, Tourcoing, France; 9 Service de Médecine Intensive Réanimation, Centre Hospitalier Départemental de La Vendée, La Roche sur Yon, France; 10 Groupe Hospitalier Paris Centre-Université de Paris, Cochin University Hospital, Medical Intensive Care Unit, Paris, France; 11 INSERM U995, Lille Inflammation Research International Center E2, Lille University, Lille, France

**Keywords:** COPD, Intubation, Mechanical ventilation, Microaspiration, Pneumonia

## Abstract

**Background:**

Although COPD patients are at higher risk for aspiration when breathing spontaneously, no information is available on the risk for microaspiration in invasively ventilated COPD patients. The aim of our study was to determine the relationship between COPD and abundant microaspiration in intubated critically ill patients.

**Methods:**

This was a retrospective analysis of prospectively collected data, provided by 3 randomized controlled trials on microaspiration in critically ill patients receiving invasive mechanical ventilation for more than 48 h. Abundant microaspiration was defined as the presence of pepsin and or alpha-amylase at significant levels in tracheal aspirates. In all study patients, pepsin and alpha-amylase were quantitatively measured in all tracheal aspirates collected during a 48-h period. COPD was defined using spirometry criteria.

**Results:**

Among the 515 included patients, 70 (14%) had proven COPD. Pepsin and alpha-amylase were quantitatively measured in 3873 and 3764 tracheal aspirates, respectively. No significant difference was found in abundant microaspiration rate between COPD and non-COPD patients (62 of 70 patients (89%) vs 366 of 445 (82%) patients, *p* = 0.25). Similarly, no significant difference was found in abundant microaspiration of gastric contents (53% vs 45%, *p* = 0.28), oropharyngeal secretions (71% vs 71%, *p* = 0.99), or VAP (19% vs 22%, *p* = 0.65) rates between the two groups. No significant difference was found between COPD and non-COPD patients in duration of mechanical ventilation, ICU length of stay, or ICU mortality.

**Conclusions:**

Our results suggest that COPD is not associated with increased risk for abundant microaspiration in intubated critically ill patients.

## Background

Ventilator-associated pneumonia (VAP) is a common ICU-acquired infection in patients requiring intubation and mechanical ventilation [1, 2]. This infection is associated with increased morbidity, mortality, and cost [3–6]. Microaspiration of gastric and oropharyngeal secretions is the first route of entry of bacteria into the lower respiratory tract in patients receiving invasive mechanical ventilation [7, 8]. Microaspiration is diagnosed in 50 to 75% of intubated patients and is related to the presence of the tracheal tube, which prevents closure of vocal cords [9–12]. Other risk factors for microaspiration include enteral nutrition, mechanical ventilation, and other patient-related factors. Tracheobronchial colonization could progress to ventilator-acquired tracheobronchitis (VAT) and VAP, when the quantity and virulence of bacteria are high, and local and general host defense are altered [13].

In spite of increased use of non-invasive ventilation in chronic obstructive pulmonary disease (COPD) patients, the proportion of COPD requiring invasive mechanical ventilation in the ICU is still high [14, 15]. Based on the results of the largest international epidemiological study on mechanical ventilation, the proportion of COPD in patients receiving invasive mechanical ventilation slightly decreased from 10% in 1998 to 7% in 2016 [16]. However, another recent large international study reported that COPD patients represented 22% of patients receiving mechanical ventilation for acute respiratory distress syndrome (ARDS) in the ICU [17].

Based on recent data, spontaneously breathing COPD patients could be at higher risk for microaspiration, because of gastro-esophageal reflux, discoordination between breathing and swallowing, cricopharyngeal muscle dysfunction, and changes in lung volume [18]. To our knowledge, no study to date has evaluated the relationship between COPD and microaspiration in intubated critically ill patients. Therefore, we conducted this retrospective analysis of prospectively collected data to determine the impact of COPD on abundant microaspiration. Secondary objectives were to determine the impact of COPD on VAP incidence, duration of mechanical ventilation, ICU length of stay, and mortality rate.

## Patients and methods

This was a retrospective analysis of prospectively collected data coming from three randomized controlled open-label studies on microaspiration in intubated critically ill patients [12, 19, 20]. The Nosten trial (single center, 122 patients) evaluated the impact of continuous control, as compared to manually intermittent control, of tracheal cuff pressure on the incidence of microaspiration [19]. The BestCuff study (multicenter, 326 patients) evaluated the impact of tapered-cuff shape, as compared to standard cuff shape, on microaspiration [20]. The ancillary study of NutriRéa-2 trial (multicenter, 151 patients) evaluated the impact of enteral nutrition, as compared to parenteral, on microaspiration in patients with septic shock [12]. This study was approved by the local Institutional Review Board (Comité de Protection des Personnes Nord Ouest IV IORG0009553, validation n° HP 20/40). In accordance with the French law, and because of the retrospective observational design, written informed consent was not required.

### Study patients

In all studies, patients were included if they were older than 18 years and if the estimated duration of invasive mechanical ventilation was more than 48 h. Polyvinyl chloride (PVC) tracheal tubes were used in all patients. Tracheal cuff shape was standard in all patients, except those randomized in tapered-cuff arm of the BestCuff study. All patients were positioned in semirecumbent position during their period of mechanical ventilation. Oral care was performed using chlorhexidine 0.1%. Tracheal cuff pressure was manually monitored using a manometer and adjusted around 25 cm H_2_O, three times a day (except in the intervention group of the Nosten study, in which cuff pressure was continuously adjusted using a pneumatic device). Subglottic secretion drainage was not used in study patients.

In the three trials, after randomization all tracheal aspirates were collected for 48 h for pepsin and alpha-amylase measurements. Tracheal aspirates were performed according to the need of each individual patient, as determined by nurses at bedside. All tracheal aspirates were stored at − 20 °C and sent to the central laboratory at Lille University Hospital, where all measurements were blindly performed (ELISA technique for pepsin and difference between total and pancreatic amylase activity for salivary amylase) [20, 21].

### Definitions

Abundant microaspiration was defined as the presence of abundant gastric contents microaspiration and/or abundant oropharyngeal secretions microaspiration. Abundant microaspiration of gastric content was defined by the presence of pepsin at significant concentration (> 200 ng/ml) in more than 30% of tracheal aspirates [20]. Abundant microaspiration of oropharyngeal secretions was defined by the presence of alpha-amylase at significant concentration (> 1685 IU/ml) in more than 30% of tracheal aspirates [20].

VAP was defined using clinical, radiographic, and microbiological criteria, namely, a new and persistent infiltrate on chest X-ray associated with two of the three following criteria: purulent tracheal aspirates, hyperthermia > 38 °C, or hypothermia < 36 °C, and peripheral leukocytosis > 10 G/l or < 1.5 G/l. In addition, microbiological confirmation was required, using tracheal aspirate ≥ 10^5^ CFU/ml or bronchoalveolar lavage ≥ 10^4^ CFU/ml [22].

COPD was defined using spirometry, by the presence of a forced expiratory volume in 1 s (FEV1)/forced vital capacity (FVC) below 70% after bronchodilators [23].

Primary outcome was the incidence of abundant microaspiration. Secondary outcomes were abundant microaspiration of gastric secretions, abundant microaspiration of oropharyngeal secretions, rate of VAP, duration of mechanical ventilation, ICU length of stay, and ICU mortality.

### Data collection

All data were prospectively collected, except those related to COPD (most recent FEV1/FVC, most recent FEV1, GOLD classification, long-term oxygen therapy, and specific respiratory medications). Patient characteristics were collected at ICU admission, during the 48 h following randomization, and during ICU stay.

### Statistical analysis

Categorical variables were expressed as numbers (percentages) and compared using Chi-square test or Fisher’s exact test, as appropriate. Normality of distribution of continuous variables was checked graphically and by using the Shapiro–Wilk test. As all quantitative variables were skewed, they are presented as medians (interquartile ranges) and compared between COPD and non-COPD patients using Mann–Whitney U test or Kruskal–Wallis test.

Univariate and multivariate analyses were performed to determine factors associated with abundant microaspiration. All variables with a *p*-value < 0.1 in univariate analysis were included in a logistic regression model using a stepwise backward elimination. Potential interactions were tested. Goodness-of-fit of the final multivariate analysis model was tested using the Hosmer and Lemeshow test.

All statistical tests were two tailed, and *p* values < 0.05 were considered statistically significant. The SPSS software package (IBM, SPSS statistics 22) was used for statistical analyses.

## Results

**Fig. 1:**
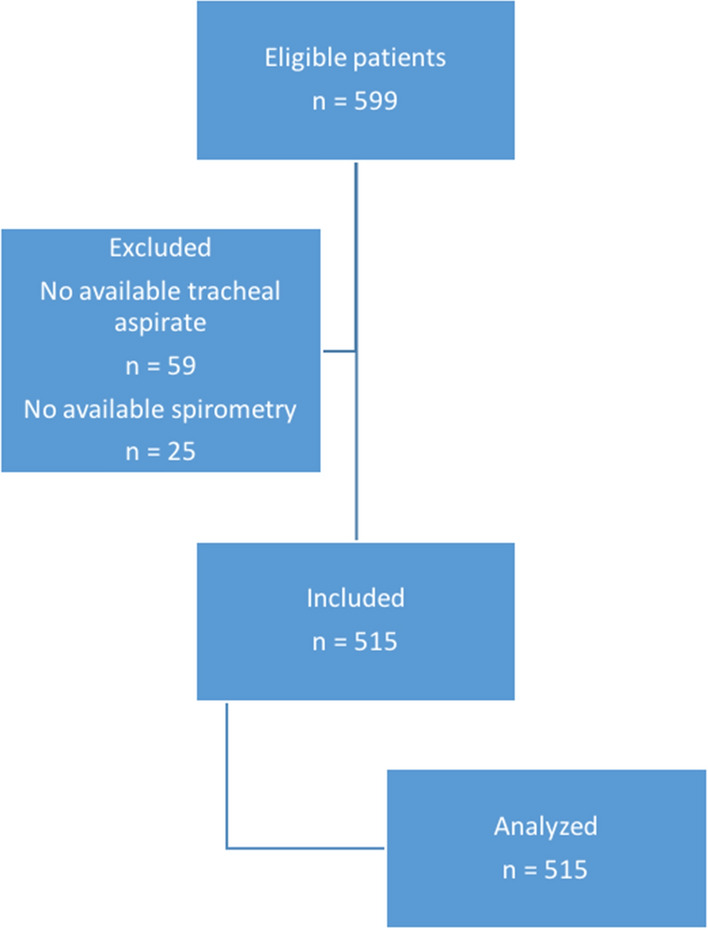
Flowchart

Five hundred ninety-nine patients were included in the three randomized controlled trials, and were eligible for this study. Eighty-four (14%) patients were excluded, including 59 (10%) because no tracheal aspirate was available for measurement of pepsin or alpha-amylase and 25 (4%) because spirometry was not available to confirm COPD diagnosis. In total, 515 patients were included and analyzed (104 from the Nosten trial, 286 from the BestCuff trial, and 125 from the Nutri-Réa-2 ancillary trial) (Fig. 1). Seventy patients (14%) had a proven COPD. Pepsin and alpha-amylase were quantitatively measured in 3873 and 3764 tracheal aspirates, respectively.

### Patient characteristics

At ICU admission, several significant differences were found between COPD and non-COPD patients (Table 1). Patients with COPD were older, more frequently male, and had more comorbidities than non-COPD patients. On the other hand, simplified acute physiology score II was significantly lower in COPD than in non-COPD patients. Causes for ICU admission also significantly differed between the two groups. The median number (IR) of tracheal aspirates used for pepsin and alpha-amylase measurements was not significantly different between COPD and non-COPD patients (8 (4, 11) vs 9 (5, 13), *p* = 0.43 and 8 (4, 11) vs 9 (5, 13), *p* = 0.74; respectively).

**Table 1:** Patient characteristics at ICU admission

	COPD		*p* value
	Yes (*n* = 70)	No (*n* = 445)	
Age (years)	67 [60–74]	62 [51–72]	0.012
Male gender	58 (83)	228 (51)	0.040
SAPS II	45 (34, 57)	51 (40, 63)	0.039
Location before ICU admission			0.12
Home	45 (64)	228 (51)	
Ward	23 (33)	201 (45)	
Other ICUs	2 (3)	16 (4)	
McCabe score			0.002
1	30 (43)	288 (65)	
2	33 (47)	129 (29)	
3	7 (10)	28 (6)	
Comorbidities			
Diabetes mellitus	22 (31)	109 (24)	0.22
Chronic cardiac failure	24 (34)	79 (18)	0.002
Cirrhosis	1 (1)	38 (9)	0.06
Chronic renal failure	7 (10)	39 (9)	0.91
Gastro-esophageal reflux	3 (4)	25 (6)	0.86
Immunosuppression	8 (11)	79 (18)	0.25
Cause for ICU admission			< 0.001
Acute respiratory failure	50 (71)	155 (35)	
Acute respiratory distress syndrome	5 (7)	52 (12)	
Shock	11 (16)	105 (24)	
Pneumonia	14 (20)	67 (15)	
Congestive heart failure	0 (0)	6 (1)	
Neurological failure	3 (4)	88 (20)	
Intoxication	1 (1)	16 (4)	
Trauma	0 (0)	5 (1)	
Post-operative	0 (0)	9 (2)	
Cardiac arrest	0 (0)	22 (5)	
Other	2 (3)	20 (4)	

Data are median (IQ), or number (%)

COPD, chronic obstructive pulmonary disease; SAPS, simplified acute physiology score; ICU, intensive care unit

Tracheal tube size and the proportion of patients who received continuous control of tracheal cuff pressure were significantly higher in COPD as compared to non-COPD patients. However, positive expiratory pressure (PEEP) was lower in COPD as compared to non-COPD patients (Table 2).

**Table 2:** Patient characteristics at randomization and during the first 48 h after intubation

	COPD		*p* value
	Yes (*n* = 70)	No (*n* = 445)	
Size of tracheal tube			0.012
6.5 and 7 mm	4 (6)	63 (14)	
7.5 mm	29 (41)	233 (52)	
8 and 8.5 mm	35 (50)	125 (28)	
Shape of tracheal cuff			0.16
Standard	51 (73)	296 (67)	
Tapered	19 (27)	149 (33)	
Continuous control of cuff pressure	14 (20)	40 (9)	0.010
Positive end expiratory pressure, H_2_O	7 (6, 9)	8 (6, 10)	0.050
Enteral nutrition	63 (90)	360 (81)	0.093
Vomiting	9 (13)	59 (13)	0.99
Prokinetics	8 (11)	50 (11)	0.99
Proton pump inhibitor	40 (57)	255 (51)	0.99
Sedation	56 (80)	356 (80)	0.99
Neuromuscular-blocking agents	14 (20)	104 (23)	0.64
Glasgow coma score	14 (7, 15)	14 (7, 15)	0.95
Ramsay score	4 (3, 4)	4 (3, 4)	0.833

Data are median (IQ), or number (%)

In COPD patients, median (IR) FEV1 was 1.30L (0.87, 1.68) (% of theoretical FEV1 46% (29, 57)), 49 of 70 (70%) patients received inhaled corticosteroids, 59 (84%) received bronchodilators, and 22 (31%) received oxygen therapy at home. Gold stages 1, 2, 3, and 4 were present in 5 (7%), 26 (37%), 16 (23%), and 23 (33%) patients; respectively.

### Outcomes

No significant difference was found in the proportion of abundant microaspiration between COPD and non-COPD patients (89% vs 82%, *p* = 0.25, Table 3). Abundant gastric and abundant oropharyngeal microaspiration did not differ between the two groups. There was no statistical difference between COPD and non-COPD groups in VAP (19% vs 22%, *p* = 0.65) or ICU mortality (47% vs 26%, *p* = 0.97) rates. Similarly, no significant difference was found in duration of mechanical ventilation, and ICU length of stay between COPD and non-COPD patients.

**Table 3:** Primary and secondary outcomes

	COPD		*p* value
	Yes (*n* = 70)	No (*n* = 445)	
Primary outcome			
Abundant microaspiration	62 (89)	366 (82)	0.25
Secondary outcomes			
Abundant microaspiration of gastric contents	37 (53)	201 (45)	0.28
Abundant microaspiration of oropharyngeal secretions	50 (71)	318 (71)	0.99
Ventilator-associated pneumonia	13 (19)	97 (22)	0.65
Duration of mechanical ventilation, d	11 (6, 18)	8 (5, 17)	0.39
ICU length of stay, d	14 (8, 23)	13 (8, 23)	0.50
ICU mortality	17 (24)	124 (28)	0.63

Data are median (IQ), or number (%)

Univariate analysis identified age, PEEP, enteral nutrition, sedation, neuromuscular-blocking agent use, and coma Glasgow score as factors significantly associated with abundant microaspiration. The only independent risk factor for abundant microaspiration was coma Glasgow score (0.96 (0.92–0.99) per point, *p* = 0.025). COPD remained not significantly (*p* = 0.40) associated with microaspiration, when it was forced in the final model (Additional file 1: Table S1).

## Discussion

Our results suggest that COPD is not associated with increased risk for abundant microaspiration in intubated critically ill patients. Proportion of patients with VAP, duration of mechanical ventilation, ICU length of stay, and mortality did not significantly differ between COPD and non-COPD patients.

To the best of our knowledge, our study is the first to evaluate the relationship between COPD and microaspiration in intubated critically ill patients. Strengths of our study are large number of included patients, multicenter design, quantitative assessment of two validated markers of microaspiration in a large number of tracheal aspirates, requirement of strict quantitative microbiological criteria for VAP diagnosis, and inclusion of only spirometry-confirmed COPD patients.

The absence of significant relationship between COPD and abundant microaspiration could be explained by high baseline rate of microaspiration, and the fact that risk factors related to intubation, mechanical ventilation, enteral nutrition through a nasogastric tube are probably more important than those only related to COPD. Zheng et al. [18] recently compared the incidence of silent aspiration, confirmed using technetium-99 m–sulfur colloid scintigraphy, between patients with acute exacerbation of COPD and non-COPD healthy volunteers. The incidence of aspiration was significantly higher in COPD than in controls (14 out of 42 *versus* 0 out of 13; *p* = 0.024). Several explanations were suggested for this result, including dyspnea, dysphagia, emphysema, abnormal swallowing function, cricopharyngeal muscle impairment, decreased throat sensitivity, and impaired cough reflex [24–26]. However, in intubated critically ill patients, most of these factors are present in COPD and non-COPD patients.

The use of pepsin and alpha-amylase as markers for microaspiration was recently validated and allows an easy and accurate evaluation of microaspiration in intubated critically ill patients [21]. However, these markers were not validated using the gold standard, i.e., the use of radioactive isotope-labeled enteral feeding, which is now restricted to radiology department [7].

The absence of significant difference in VAP rate between COPD and non-COPD patients is in line with the absence of significant difference in abundant microaspiration rate between the two groups. Recent large multicenter studies reported that COPD was not significantly associated with increased risk for VAP [27, 28]. In a planned post hoc analysis of the TAVeM international large multicenter study, our group found similar rates of VAP in COPD and non-COPD patients (12% versus 13%, *p* = 0.93) [28]. Other older studies identified COPD as an independent risk factor for VAP [29, 30]. However, in all these studies COPD definition was not based on spirometry findings.

No significant difference was found in duration of mechanical ventilation, ICU length of stay, or mortality between COPD and non-COPD patients receiving mechanical ventilation for more than 48 h. This result is also in line with recent findings [28], highlighting the fact that COPD patients should be admitted to the ICU when necessary, and all therapeutic measures should be used.

Our study has several limitations: First, it was retrospective. However, all data, except those related to COPD diagnosis and severity, were prospectively collected. Second, the number of patients with COPD was relatively small. However, the percentage of COPD in study patients (14%) is in line with previous findings of large international cohorts [16, 17]. In addition, 25 patients were excluded because spirometry was not available. However, similar results were found when these patients were included in outcome analyses (Additional file 1: Table S2). Further, no information was available on the delay between spirometry and ICU admission, and spirometry was not available for patients with no suspected COPD. However, it seems unlikely to have misclassified COPD patients in the non-COPD group, as all patients with suspected COPD were excluded. Third, compliance with microaspiration prevention measures was not evaluated and no information was available on some risk factors for microaspiration, such as head-of-bed elevation, tracheal cuff pressure, or total PEEP. COPD patients are prone to develop intrinsic PEEP due to chronic bronchial obstruction, resulting in dynamic hyperinflation. Fourth, tracheal suctioning was performed based on patient’s need, and subglottic secretion drainage was not performed in study patients, which might have influenced the results. Finally, several significant differences were found between COPD and non-COPD patients, such as the more frequent use of continuous control of cuff pressure in COPD patients. Similarly, the size of tracheal tube was higher in COPD than in non-COPD patients. This is probably related to the fact that male gender was significantly higher in COPD patients. However, similar results were found in multivariate analysis when COPD was forced in the final model.

In conclusion, our results suggest that COPD is not a risk factor for abundant microaspiration, or VAP. Further, COPD had no significant impact on duration of mechanical ventilation, ICU length of stay, or ICU mortality. Therefore, no specific preventive measures for microaspiration or VAP are needed in COPD patients receiving invasive mechanical ventilation, as compared to non-COPD patients. Further studies should focus on COPD patients requiring non-invasive mechanical ventilation and/or high flow nasal oxygen and determine the impact of microaspiration on ICU-acquired pneumonia in this population.

## Supplementary Information

**Additional file 1: Table S1.** Risk factors for abundant microaspiration by univariate and multivariate analyses. **Table S2.** Outcomes of patients, including suspected COPD.

## Data Availability

All data are provided in the manuscript.

## Abbreviations

ARDS: Acute respiratory distress syndrome
CI: Confidence interval
COPD: Chronic obstructive pulmonary disease
FEV_1_: Forced expiratory volume in 1 s
FVC: Forced vital capacity
ICU: Intensive care unit
IR: Interquartile range
PEEP: Positive end expiratory pressure
VAP: Ventilator-associated pneumonia

## References

[1] Nair GB, Niederman MS (2014). Ventilator-associated pneumonia: present understanding and ongoing debates. Intensive Care Med.

[2] Papazian L, Klompas M, Luyt C-E (2020). Ventilator-associated pneumonia in adults: a narrative review. Intensive Care Med.

[3] Bekaert M, Timsit JF, Vansteelandt S (2011). Attributable mortality of ventilator-associated pneumonia: A reappraisal using causal analysis. Am J Respir Crit Care Med.

[4] Melsen WG, Rovers MM, Groenwold RHH (2013). Attributable mortality of ventilator-associated pneumonia: A meta-analysis of individual patient data from randomised prevention studies. Lancet Infect Dis.

[5] Safdar N, Dezfulian C, Collard HR, Saint S (2005). Clinical and economic consequences of ventilator-associated pneumonia: a systematic review. Crit Care Med.

[6] Nseir S, Di PC, Soubrier S (2005). Impact of ventilator-associated pneumonia on outcome in patients with COPD. Chest.

[7] Nseir S, Zerimech F, Jaillette E, Artru F, Balduyck M (2011). Microaspiration in intubated critically ill patients: diagnosis and prevention. Infect Disord Drug Targets.

[8] Nseir S, Ader F, Lubret R, Marquette C-H (2011). Pathophysiology of airway colonization in critically ill COPD patient. Curr Drug Targets.

[9] Metheny NA, Clouse RE, Chang Y-H, Stewart BJ, Oliver DA, Kollef MH (2006). Tracheobronchial aspiration of gastric contents in critically ill tube-fed patients: frequency, outcomes, and risk factors. Crit Care Med.

[10] Monsel A, Lu Q, Le CM (2016). Tapered-cuff endotracheal tube does not prevent early postoperative pneumonia compared with spherical-cuff endotracheal tube after major vascular surgery: a randomized controlled trial. Anesthesiology.

[11] Millot G, Boddaert P, Parmentier-Decrucq E (2018). Impact of subglottic secretion drainage on microaspiration in critically ill patients: a prospective observational study. Ann Transl Med.

[12] Nseir S, Le GA, Lascarrou J-B (2019). Impact of nutrition route on microaspiration in critically ill patients with shock: a planned ancillary study of the NUTRIREA-2 trial. Crit Care.

[13] Rouzé A, Jaillette E, Nseir S (2018). Relationship between microaspiration of gastric contents and ventilator-associated pneumonia. Ann Transl Med.

[14] Rouzé A, Cottereau A, Nseir S (2014). Chronic obstructive pulmonary disease and the risk for ventilator-associated pneumonia. Curr Opin Crit Care.

[15] Makris D, Desrousseaux B, Zakynthinos E, Durocher A, Nseir S (2011). The impact of COPD on ICU mortality in patients with ventilator-associated pneumonia. Respir Med.

[16] Penuelas O, Muriel A, Abraira V (2020). Inter-country variability over time in the mortality of mechanically ventilated patients. Intensive Care Med.

[17] Bellani G, Laffey JG, Pham T (2016). Epidemiology, patterns of care, and mortality for patients with acute respiratory distress syndrome in intensive care units in 50 countries. JAMA.

[18] Zheng Z, Wu Z, Liu N (2016). Silent aspiration in patients with exacerbation of COPD. Eur Respir. J..

[19] Nseir S, Zerimech F, Fournier C (2011). Continuous control of tracheal cuff pressure and microaspiration of gastric contents in critically ill patients. Am J Respir Crit Care Med.

[20] Jaillette E, Girault C, Brunin G (2017). Impact of tapered-cuff tracheal tube on microaspiration of gastric contents in intubated critically ill patients: a multicenter cluster-randomized cross-over controlled trial. Intensive Care Med.

[21] Dewavrin F, Zerimech F, Boyer A (2014). Accuracy of alpha amylase in diagnosing microaspiration in intubated critically-ill patients. PLoS ONE.

[22] Guidelines for the management of adults with hospital-acquired (2005). ventilator-associated, and healthcare-associated pneumonia. Am J Respir Crit Care Med.

[23] Global Initiative for Chronic Obstructive Lung Disease Global Initiative for Chronic Obstructive Lung Disease. A Guide for Health Care Professionals. 2019. www.goldcopd.org; accessed August 18, 2020

[24] Stein M, Williams AJ, Grossman F, Weinberg AS, Zuckerbraun L (1990). Cricopharyngeal dysfunction in chronic obstructive pulmonary disease. Chest.

[25] Gross RD, Atwood CW, Ross SB, Olszewski JW, Eichhorn KA (2009). The coordination of breathing and swallowing in chronic obstructive pulmonary disease. Am J Respir Crit Care Med.

[26] Mandell LA, Niederman MS (2019). Aspiration Pneumonia. N Engl J Med.

[27] Koulenti D, Blot S, Dulhunty JM (2015). COPD patients with ventilator-associated pneumonia: implications for management. Eur J Clin Microbiol Infect Dis.

[28] Rouzé A, Boddaert P, Martin-Loeches I (2020). Impact of chronic obstructive pulmonary disease on incidence, microbiology and outcome of ventilator-associated lower respiratory tract infections. Microorganisms.

[29] Torres A, Aznar R, Gatell JM (1990). Incidence, Risk, and Prognosis Factors of Nosocomial Pneumonia in Mechanically Ventilated Patients. Am Rev Respir Dis.

[30] Tejerina E, Frutos-Vivar F, Restrepo MI (2006). Incidence, risk factors, and outcome of ventilator-associated pneumonia. J Crit Care.

