# Supplementary material for: Is COPD associated with increased risk for microaspiration in intubated critically ill patients?
Source: Ann Intensive Care. 2021 Jan 11;11:7. doi: 10.1186/s13613-020-00794-1 (PMC7798009; doi:10.1186/s13613-020-00794-1)
Supplement: Supplementary file 1 — Additional file 1: Table S1. Risk factors for abundant microaspiration by univariate and multivariate analyses. Table S2. Outcomes of patients, including suspected COPD. [file 13613_2020_794_MOESM1_ESM.docx]

**Additional file 1: Table S1. Risk factors for abundant microaspiration by univariate and multivariate analyses**

| Factors | Univariate analysis | | | Multivariate analysis | |
| --- | --- | --- | --- | --- | --- |
|  | Abundant microaspiration  n = 428 | No abundant microaspiration  n = 87 | P value | OR (95% CI) | P value |
| At ICU admission |  |  |  |  |  |
| Age | 65 (54, 74) | 58 (56, 71) | 0.003 | - | - |
| Mc Cabe score |  |  | 0.074 | - | - |
| 1 | 267 (62) | 51 (59) |  |  |  |
| 2 | 128 (30) | 34 (39) |  |  |  |
| 3 | 33 (8) | 2 (2) |  |  |  |
| Chronic cardiac failure | 92 (21) | 11 (13) | 0.083 | - | - |
| During the 48h following randomization |  |  |  |  |  |
| Positive end expiratory pressure, mean±SD | 7.8±3.6 | 6.6±2.6 | 0.006 | 0.94 (0.87-1.10) | 0.071 |
| Enteral nutrition | 359 (84) | 69 (79) | 0.033 | - | - |
| Sedation | 336 (79) | 76 (87) | 0.083 | - | - |
| Neuromuscular blocking agent use | 81 (19) | 37 (43) | <0.001 | 0.67 (0.42-1.06) | 0.088 |
| Glasgow coma score | 13 (6, 15) | 15 (11, 15) | <0.001 | 0.96 (0.92-0.99) | 0.025 |
| COPD | 62 (14) | 8 (9) | 0.25 | 1.26 (0.73-2.18) | 0.40 |

OR, odds ratio; CI, confidence interval; COPD, chronic obstructive pulmonary disease.

Data are N° (%) or median (IR), unless otherwise specified.

All other factors presented in Tables 1, and 2 were not significantly associated (P>0.1) with abundant microaspiration by univariate analysis.

Hosmer and Lemshow goodness-of-fit test P = 0.87

**Additional file 1: Table S2. Outcomes of patients, including suspected COPD**

|  | Suspected COPD | | P value |
| --- | --- | --- | --- |
|  | Yes (n = 95) | No (n = 445) |  |
| Primary outcome |  |  |  |
| Abundant microaspiration | 85 (89) | 366 (82) | 0.12 |
| Secondary outcomes |  |  |  |
| Abundant microaspiration of gastric contents | 52 (55) | 201 (45) | 0.11 |
| Abundant microaspiration of oropharyngeal secretions | 72 (76) | 318 (71) | 0.47 |
| Ventilator-associated pneumonia | 18 (19) | 97 (22) | 0.63 |
| Duration of mechanical ventilation, d | 9 (5, 14) | 8 (5, 17) | 0.98 |
| ICU length of stay, d | 13 (8, 22) | 13 (8, 23) | 0.99 |
| ICU mortality | 24 (25) | 124 (28) | 0.69 |

Data are median (IQ), or number
